# EhCoactosin Stabilizes Actin Filaments in the Protist Parasite *Entamoeba histolytica*

**DOI:** 10.1371/journal.ppat.1004362

**Published:** 2014-09-11

**Authors:** Nitesh Kumar, Somlata, Mohit Mazumder, Priyanka Dutta, Sankar Maiti, Samudrala Gourinath

**Affiliations:** 1 School of Life Sciences, Jawaharlal Nehru University, New Delhi, India; 2 Indian Institute of Science Education and Research, Kolkata, India; University of Virginia Health System, United States of America

## Abstract

*Entamoeba histolytica* is a protist parasite that is the causative agent of amoebiasis, and is a highly motile organism. The motility is essential for its survival and pathogenesis, and a dynamic actin cytoskeleton is required for this process. EhCoactosin, an actin-binding protein of the ADF/cofilin family, participates in actin dynamics, and here we report our studies of this protein using both structural and functional approaches. The X-ray crystal structure of EhCoactosin resembles that of human coactosin-like protein, with major differences in the distribution of surface charges and the orientation of terminal regions. According to *in vitro* binding assays, full-length EhCoactosin binds both F- and G-actin. Instead of acting to depolymerize or severe F-actin, EhCoactosin directly stabilizes the polymer. When EhCoactosin was visualized in *E. histolytica* cells using either confocal imaging or total internal reflectance microscopy, it was found to colocalize with F-actin at phagocytic cups. Over-expression of this protein stabilized F-actin and inhibited the phagocytic process. EhCoactosin appears to be an unusual type of coactosin involved in *E. histolytica* actin dynamics.

## Author Summary

*E. histolytica* is an important pathogen and a major cause of morbidity and mortality in developing nations. High level of motility and phagocytosis is responsible for the parasite invading different tissues of the host. Phagocytosis and motility depend on highly dynamic actin cytoskeleton of this organism. The mechanisms of actin dynamics is not well understood in *E. histolytica*. Here we report that coactosin like molecule from *E. histolytica*, EhCoactosin is involved in F-actin stabilization. The crystal structure obtained for the protein provides explanation for some functional differences observed with respect to the human homologue, such as ability to bind G-actin. Moreover, computational modelling along with crystal structure helps to explain the F-actin binding and stabilization by wild type protein. The mutational analysis further suggests that F-actin binding property does not depend on conserved Lys^75^ residue as observed in Human coactosin like protein (HCLP) but other regions present in protein are involved in binding. Overexpression of this protein in trophozoites leads to stabilization of actin filaments which are not accessible to actin remodelling machinery thereby reducing the growth of parasite due to decreased rate of actin dependent endocytosis. Overall, EhCoactosin behaves as F-actin stabilizing protein *in vitro* and it also participates in processes like phagocytosis and pseudopod formation.

## Introduction

Human amoebiasis is caused by the protist parasite *E. histolytica.* The parasite is highly motile and displays high level of phagocytic activity in the trophozoite stage. Motility and phagocytosis are essential processes for the survival and invasion of host tissues by the parasite, and largely depends on a highly dynamic actin cytoskeleton. Moreover, there are other processes, such as phagocytosis that also require dynamic actin filament reorganization. Molecular mechanisms that regulate actin dynamics in *E. histolytica* have not been studied in detail. Preliminary investigations suggest an overall similarity with those described in other eukaryotic cells, but with crucial differences. For example, a number of calcium-sensing calcium-binding proteins appear to directly regulate actin recruitment and dynamics [1], [2], [3]. Several actin-binding proteins are encoded by the *E. histolytica* genome and many of these proteins are homologs of those that have been studied in other systems. Not many of these amebic actin-binding proteins have been characterized. Understanding structural-functional relationship of these proteins would help to decipher mechanisms of actin dynamics in *E. histolytica*.

In *E. histolytica* as well as many other cells, actin dynamics involves both assembly and disassembly of filaments regulated by several actin-binding proteins. The actin-binding protein coactosin was first identified in *Dictyostelium discoidedeum* and has been classified as a member of actin depolymerising factor (ADF)/cofilin family [4]. The ADF/cofilin family members are expressed in all eukaryotes studied to date. The human coactosin-like protein (HCLP) binds F-actin and interferes with capping of filaments. However it does not affect actin polymerisation [5]. HCLP is also known to bind 5-lipooxygenase [6]. The binding of members of the ADF/cofilin family to the F-actin results in severing and depolymerisation of F-actin [7]. However the precise function of this family may vary from actin nucleation to severing depending on the cellular concentration gradient of cofilin [7].

The *E. histolytica* genome contains only one copy of the coactosin gene, whose product we refer to as EhCoactosin. Since the role of EhCoactosin in the actin dynamics of *E. histolytica* has not been previously investigated, we have carried out structural and functional analyses of this protein and present the results here. They show that a single conserved ADF homology domain of EhCoactosin is involved in binding F-actin, and that F-actin is stabilized when EhCoactosin is bound. Moreover, mutation of conserved lysine 75 to alanine does not result in loss of F-actin binding, in contrast to that observed in the case of HCLP, and the binding of this mutant EhCoactosin yields a similar level of F-actin stabilization as does the binding of native EhCoactosin. But deletion of complete F-loop completely abolishes G-actin binding with loss of F-actin stabilization activity, albeit still binds to F-actin. We also propose a mechanism for the binding of EhCoactosin to actin based on a structural model obtained by X-ray crystallography. Overall our results suggest that EhCoactosin displays some features not seen in coactosin from other organisms.

## Results

Motility and phagocytosis are important processes for biology of *E. histolytica* as these are involved in providing nutrition and pathogenesis. It is well known that actin dynamics is key in regulation of above mentioned processes. In *E. histolytica* not many proteins that regulate actin dynamics have been described. Our group is analysing systematically the *E. histolytica* homologs of known actin-binding proteins both functionally as well as structurally. In this article we have described *E. histolytica* homolog of coactosin like protein.

### Bioinformatic analysis of EhCoactosin

**Figure 1:**
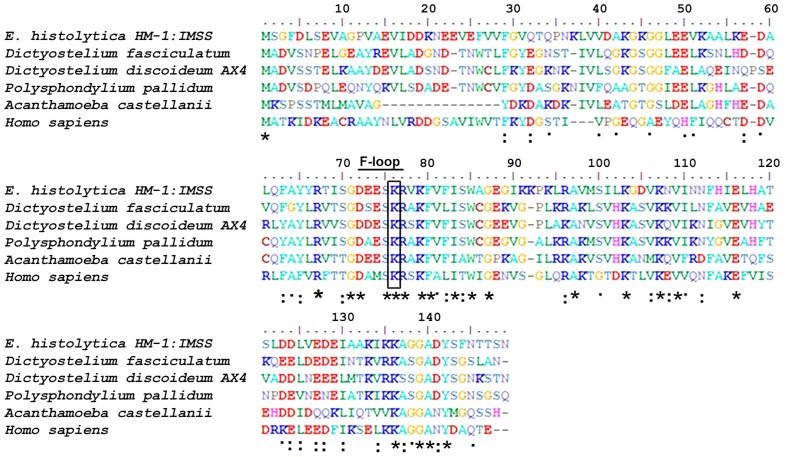
Multiple sequence alignment of coactosin-like proteins (CLPs). The sequence of EhCoactosin shares 40% identity with human CLP protein. The residues marked with asterisks are conserved, while those marked with two dots and one dot represent conservative and nonconservative substitutions, respectively.

A multiple sequence alignment of EhCoactosin [Acc No XP_650926.1 from the NCBI database] with homologous proteins from different organisms allowed us to identify numerous residues that are conserved in this family of proteins, as well as those unique to EhCoactosin (Figure 1). The amebic Coactosin sequence displays 40% similarity with both human and *D. discoidedeum* CLPs. Among the conserved residues is a critical lysine at position 75, known to be involved in F-actin binding [8].

### EhCoactosin binds to F-actin

**Figure 2:**
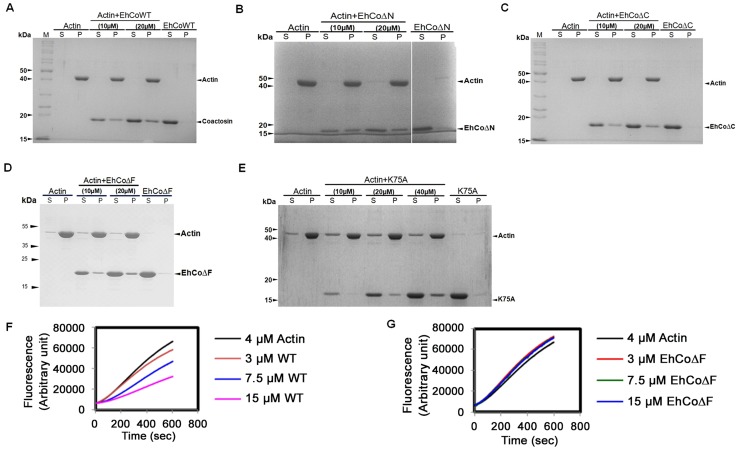
Binding of wild-type and mutant EhCoactosin proteins to F-actin by co-sedimentation. (A) Wild-type protein was found to bind actin filaments. (B) N-terminal and (C) C-terminal truncation mutants are also able to bind F-actin and sediment. (D) EhCoΔF also binds F-actin. (E) The K75A mutant shows somewhat reduced binding to F-actin, but a prominent fraction nevertheless sediments at high concentration. (F) In the G-actin binding assay, wt-EhCoactosin binds to monomeric actin florescence in dose dependent manner while (G) EhCoΔF is not able to bind the monomers and hence cannot cause reduction in florescence. S – supernatant, P- pellet.

The binding of EhCoactosin to F-actin was assessed by a sedimentation assay as described previously (1). The full-length wild-type (WT) protein binds F-actin, as it was found in the pellet fraction after ultracentrifugation (Figure 2A). A similar level of F-actin binding was also observed for truncated versions of EhCoactosin where either the N-terminal seven amino acid residues (EhCoΔN, Figure 2B) or C-terminal 14 residues (EhCoΔC, Figure. 2C) were deleted. In an attempt to narrow down the specific region involved in actin binding, we deleted the F-loop of EhCoactosin (from 71–76 amino acids) and also mutated the critical Lys^75^ residue. The F-loop deleted version of EhCoactosin (ΔF) retained actin binding property (Figure 2D). The K75A mutant of EhCoactosin was able to bind F-actin, (Figure 2E), which is in contrast to the complete loss of F-actin binding caused by the same mutation in HCLP [8]. These observations suggest that F-actin binding by EhCoactosin does not solely depend on F-loop and Lys^75^.

### EhCoactosin binds G-actin directly

G-actin binding was determined by a G-actin sequestering and solid phase assay as described previously [1]. G-actin sequestering assay uses fluorescently labelled G-actin and when a protein binds the labelled actin its florescence decreases mostly in dose dependent manner. The WT EhCoactosin shows G-actin sequestering in dose dependent manner (Figure 2F) while the EhCoΔF has no G-actin binding activity as seen in Figure 2G. We also confirmed G-actin binding for other truncated versions of proteins by this assay. EhCoΔC and EhCoΔN showed dose-dependent G-actin binding but affinity of EhCoΔN was more than EhCoΔC as 10 µM of EhCoΔN was able to sequester same amount of G-actin as 25 µM of EhCoΔC (Figure S1A and B).

EhCoactosin displays specific G-actin binding was also confirmed by binding to a plate coated with G-actin. The level of binding was 2-fold higher than that of the known G-actin-binding protein EhCaBP1 [1] (Figure S1(C)).While EhCoΔC showed a 33% decrease in binding when compared to the WT protein, EhCoΔN exhibited a 2-fold increase in G-actin binding in comparison to WT. The homolog pfADF1, which binds G-actin strongly [9] is positively charged at the N-terminal region compared to EhCoactosin.

The deletion of N-terminal residues in EhCoactosin exposes more positive charges in this region (Figure S2) which, by analogy with pfADF1, may explain the increased affinity of this mutant for G-actin. The F-loop deleted (EhCoΔF) version exhibited complete loss of G-actin binding which was also observed with G-actin sequestering assay.

### EhCoactosin stabilizes F-actin

**Figure 3:**
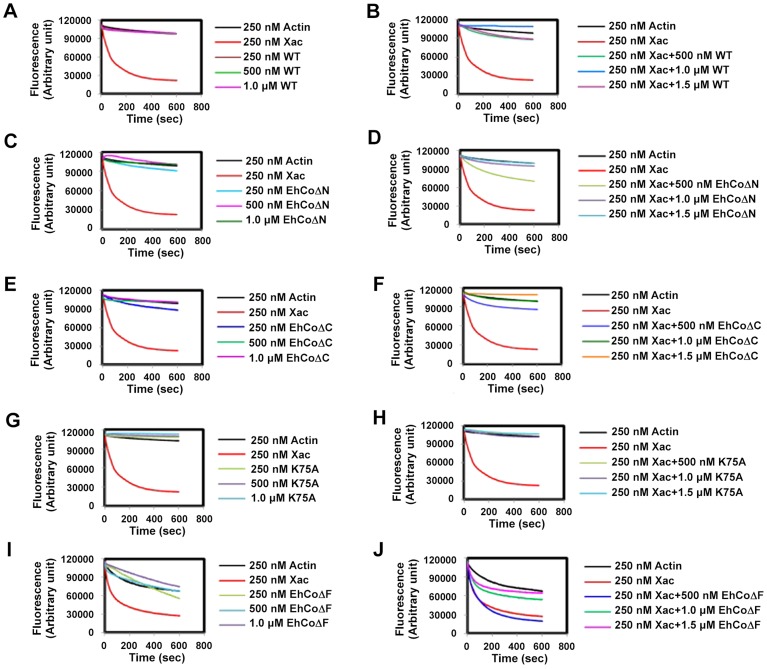
Effect of EhCoactosin on actin depolymerisation. Pyrene-labeled F-actin was used for this study and effect was assessed by the rate of decrease in fluorescence of pyrene F-actin. (A) The wt protein shows an actin stabilizing effect in comparison to actin alone. (B) Pyrene F-actin is protected from Xac1 when EhCoactosin is added. (C) EhCoΔC also has a stabilizing effect and (D) antagonizes the effect of Xac1. (E) EhCoΔN has similar stabilizing effect on F-actin as the wild type protein and (F) antagonizes the effect of Xac1. (G) and (H) K75A mutants also has similar effect. (I) the F-loop deleted protein does not stabilizes F-actin and (J) EhCoΔF is not able to protect F-actin from Xac1 activity.

The role of EhCoactosin in F-actin stabilization was determined by a pyrene-actin assay where fluorescence of pyrene-labelled F-actin decreases upon depolymerisation. The assay showed relative stabilization of F-actin by EhCoactosin compared to that by *Xenopus* cofilin1 (Xac1) (Figure 3A), and the stabilization effect was confirmed by the ability of EhCoactosin to antagonize the F-actin severing activity of Xac1 (Figure 3B) [10]. That is, while addition of Xac1 led to a sharp decrease in fluorescence, indicating its severing effect on F-actin, in the presence of EhCoactosin no decrease in fluorescence was observed and values were similar to that seen with only actin. The results suggest that EhCoactosin may be protecting F-actin from severing (Figure 3B). We also checked possibility of interaction between Xac1 and EhCoactosin by pull down assay which may lead to similar results. We found that Xac1 and EhCoactosin and its mutants do not interact directly with each other (Figure S3).

EhCoΔC and EhCoΔN showed actin stabilization similar to that of the wild-type protein (Figure 3C and 3E), and a similar stabilization effect was also observed in the case of the K75A mutant (Figure 3G). Moreover, both truncated versions and K75A mutant of EhCoactosin antagonised Xac1-dependent F-actin severing (Figure 3D, 3F and 3H). However, EhCoΔN and Xac1 at 2∶1 ratio did show mild F-actin severing (Figure 3D); the apparent weaker protection conferred by this mutant may be result of its high affinity for G-actin (Figure S1). However, the EhCoΔF had lesser F-actin stabilizing property than WT protein (Figure 3I) as in presence of the protein F-actin depolymerised to an extent. Also EhCoΔF was not able to protect F-actin from Xac1 activity (Figure 3J). These results indicate that F-loop is very essential for stable F and G-actin binding. The deletion of F-loop results in lower affinity towards F-actin making it accessible for Xac1 activity. Hence the whole F-loop plays an essential role in stable binding rather than conserved lysine residue at 75^th^ position.

### Biological importance of EhCoactosin

#### *In vitro* localisation and involvement in erythrophagocytosis

**Figure 4:**
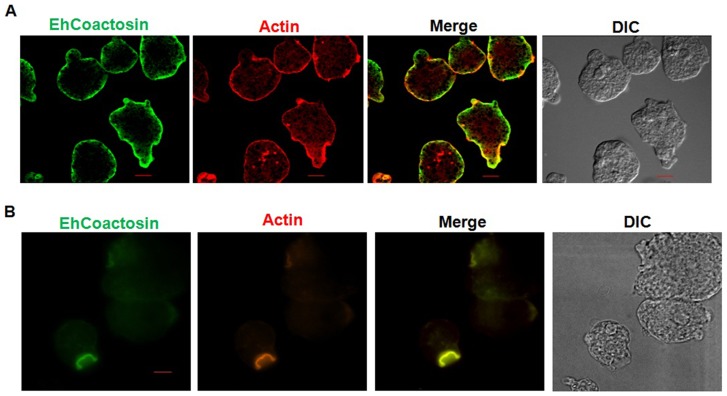
Localization of EhCoactosin in *E. histolytica* trophozoites. Immunolocalization was carried out using either an antibody against EhCoactosin (wild type) or against an HA tag using a confocal microscope. (A) wt-EhCoactosin is associated with the membrane, where it is enriched in comparison to the cytosol. (B) TIRFM revealed the presence of HA-tagged wt-EhCoactosin at phagocytic cups, where it colocalized with F-actin. Cells were undergoing phagocytosis, as RBCs were present in the system.

**Figure 5:**
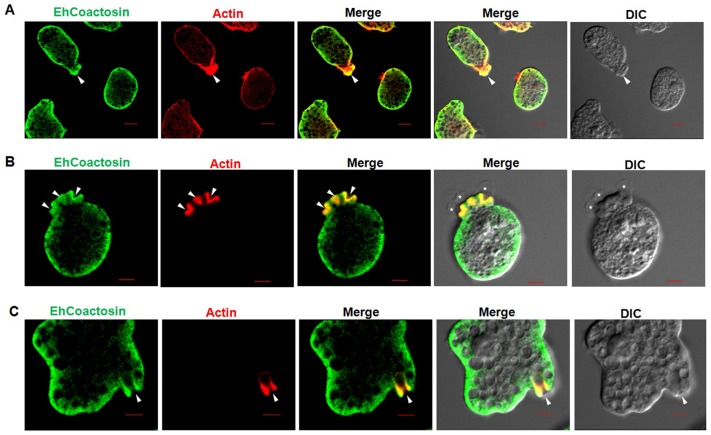
Localization of EhCoactosin in motile trophozoites undergoing erythrophagocytosis. (A) wt-EhCoactosin localized in pseudopods along with actin. (B) wt-EhCoactosin colocalizes with actin at phagocytic cups. Arrows indicate phagocytic cups and the asterisk indicates erythrocyte. (C) EhCoactosin colocalizes with F-actin in a phagocytic cup as it is about to close. EhCoactosin shows parallel distribution with F-actin. TRITC-Phalloidin was used to mark actin filaments. EhCoactosin was probed by specific antibody, HA tagged-EhCoactosin was probed by anti-mouse-HA antibody and both were visualized using Alexa488 labeled secondary antibody.

Wild-type EhCoactosin was found mainly in the plasma membrane by confocal microscopy (Figure 4A). The HA-tagged protein was also expressed in trophozoites (Figure S4) and tagged protein showed the same membrane localization as the wild-type protein. The HA-tagged protein was also visualized by Total Internal Reflectance Microscopy (TIRFM) during erythrophagocytosis. The tagged protein appeared to localize at phagocytic cups along with F-actin (Figure 4B). The protein colocalised with F-actin in pseudopods (Figure 5A) and in phagocytic cups (Figure 5B). EhCoactosin remained on the site of phagocytosis until scission of the cups was about to occur (Figure 5C). As the intensity of F-actin decreased in phagocytic cups, there was also a loss of EhCoactosin staining intensity at the sites. All these results suggest that EhCoactosin is involved in actin dynamics during erythrophagocytosis.

**Figure 6:**
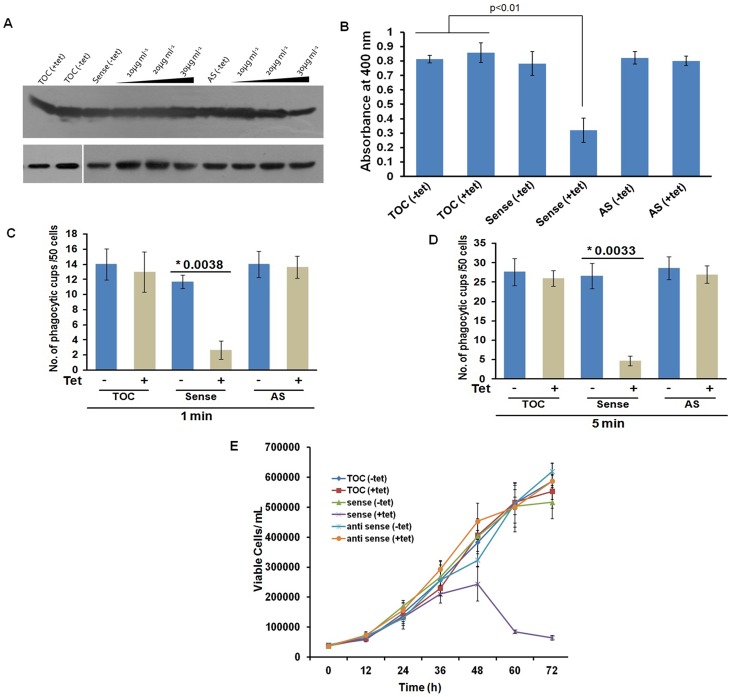
Effect of EhCoactosin over-expression on growth of trophozoites. The overexpression and underexpression of EhCoactosin in trophozoites is regulated by a tetracycline inducible promoter. (A) Immunoblot analysis of amebic cells with recombinant constructs. Cells carrying vector alone in presence and absence of tetracycline showed similar levels of EhCoactosin (lane 1 and 2 from left). Cells carrying EhCoactosin in the sense orientation showed over-expression (sense) of protein upon tetracycline induction (lane 4–6 from left) in comparison to cells with vector alone (lane 1and 2). The antisense construct (lane 7) and the vector alone (TOC) (lane 1 and 2) in the absence of tetracycline did not show significant differences in EhCoactosin levels. Induction of EhCoactosin antisense RNA in the presence of tetracycline (lanes 8–10 from left) reduced the expression of EhCoactosin in comparison to only vector containing cells in the presence of tetracycline (lane 2). The equal loading of total lysate has been shown by probing for EhCaBP1, which is not affected by inducing agent. (B) Erythrophagocytosis by trophozoites overexpressing and underexpressing EhCoactosin for fixed time of 15 min. (C) and (D) Cells with indicated constructs with and without tetracycline were incubated with human erythrocytes for indicated time points and then were fixed and stained with TRITC-Phalloidin. Initiation of phagocytosis was marked by accumulation of actin at the phagocytic cups. Fifty cells were randomly selected for each experiment and the number of phagocytic cups present in all the cells was then counted. The experiment was carried out independently thrice and statistical significance was tested by ‘t test’. The * marks the p values in figure. (E) Growth curve of *E. histolytica* trophozoites overexpressing and underexpressing EhCoactosin over 72 h. The growth is monitored in the absence and presence of tetracycline.

Furthermore, the function of EhCoactosin was investigated by either over-expressing or conditional expression blocking using tetracycline inducible whole gene antisense approach [1] (Figure S5) (Figure 6A). Trophozoites over-expressing the wild-type protein upon tetracycline induction showed 50% decrease in erythrocyte uptake as compared to that of the control (Figure 6B). When we calculated the number of cups formed, there was a reduction of about 80% (at 1 min incubation with erythrocyte) after over-expression of EhCoactosin (Figure 6C and D). However, on expression blocking no significant effect was observed (Figure 6D). It appears that an increase in the concentration of EhCoactosin above a critical limit had a profound effect on phagocytosis which is similar to stabilizing F-actin by Phalloidin and cytochalasin like drugs.

#### Effect on growth kinetics

The growth of the parasite was also monitored during over- and under-expression of the gene for 72 h. The growth declined after 48 h of tetracycline induction as compared with control cells with and without induction (Figure 6E). The surviving cells displayed altered morphology (round), low motility, and signs of stress. The expression-blocked cells showed no significant alteration in proliferation as compared to control cells in the presence and the absence of tetracycline. This experiment, along with those described above, indicate that EhCoactosin functionally stabilizes actin filaments and protects them from depolymerisation directly, which was not observed previously with homologous proteins in other systems.

### Overall structure of EhCoactosin

**Figure 7:**
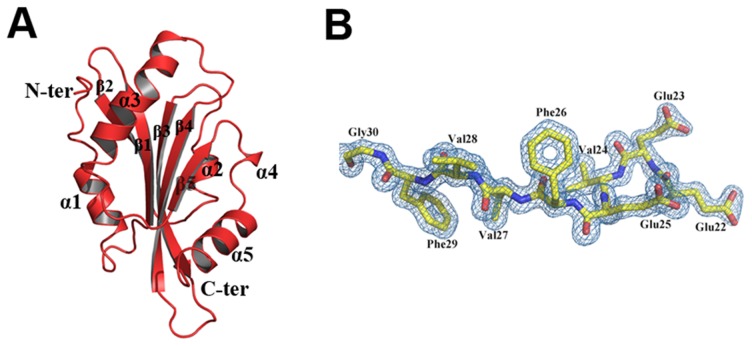
A) Crystal structure of EhCoactosin. (B) 2Fo-Fc electron density map of part of EhCoactosin at a 1.5σ cut-off.

EhCoactosin consists of a central core of β-sheets surrounded by α-helices. The central core is made up of five strands: β1(26–32), β2(37–44), β3(60–69), and β4(76–85) forming antiparallel strands while β5-strand (113–117) forms parallel strand with β3 and β4. The central β-sheets are flanked on both sides by a total of five helices; α1(9–17) and α3(92–107) are located on the N-terminal side, and α2(48–54), α4(120–122) and α5(125–137) are located on the C-terminal side (Figure 7A and B). This arrangement of secondary structural elements is a common structural feature of proteins belonging to the ADF/cofilin family.

EhCoactosin has a long N-terminal end protruding outside with Ser repeats and this signature Ser repeats is expected to bind G-actin as seen in PfADF1 [9], however wild type EhCoactosin binds to F-actin and EhCoΔN shows higher affinity for G-actin, indicating the “Ser” repeats on the N-terminal are not involved in G-actin binding. The loop connecting strands β3 and β4, which has a conserved lysine at position 75, is called the “F-loop” and it is expected to participate in stabilizing and binding to F-actin [11]. As described in more detail below, the surface of EhCoactosin is highly negatively charged, and this F-loop is part of the negatively charged surface. The N-terminal end and the F-loop are at two opposite sides of the globular structure (Figure 7) suggesting that EhCoactosin binds G-actin and F-actin in very different ways.

### Surface charge distribution of EhCoactosin is different from other known related structures

**Figure 8:**
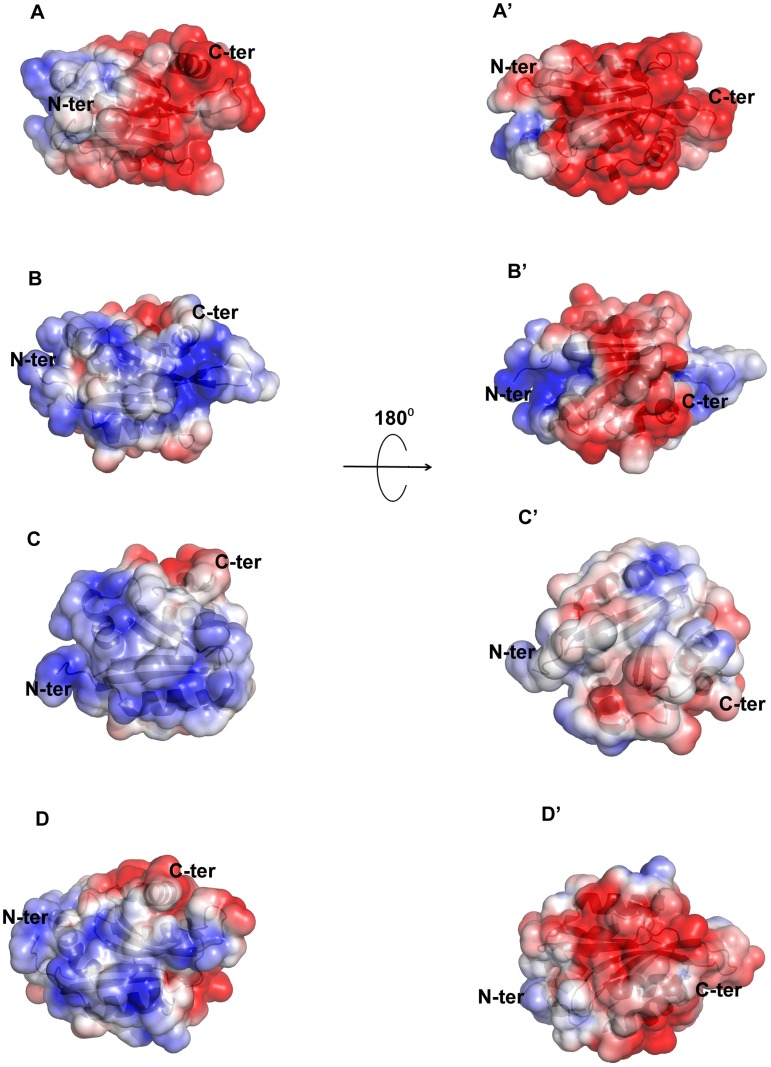
Molecular surface charge distribution of EhCoactosin and homologous proteins. The figure was prepared with Pymol. Negatively charged (red, Pymol scale of −3), positively charged (blue, Pymol scale of +3), and neutral (grey) surfaces are shown. Views of opposite sides of each protein are shown. (A,A′) The EhCoactosin surface shows a heavy distribution of negative charge, especially surrounding the C-terminus. (B, B′) One side of HCLP is predominantly positively charged, while the other side is mostly negative. Its F-loop is hydrophobic. (C, C′) In PfADF1, one side is mostly positive and the other side is hydrophobic. Its C-terminal half doesn't have an F-loop, so at this half its surface is rounder than that of the other proteins. (D,D′) In PfADF2 one side is positive and other is negative, like other ADF/Cofilin surfaces. Its C-terminal F-loop surface is negatively charged, whereas in HCLP (panel B), it is positively charged.

Although there is a general similarity of the overall conformation of EhCoactosin with that of related proteins in other organisms, the surface charge distributions of EhCoactosin is markedly distinctive. The surfaces of both sides of EhCoactosin are quite negatively charged, although one surface has overall higher level of negative charge as compared to the other surface. Just a small positively charged surface is found in the α3 and α4 region, as well as is between the β4 and α3 regions, and a hydrophobic pocket is formed between β3 and α5 (Figure 8A and 8A′). In contrast, human coactosin-like protein (HCLP) is positively charged on one side, while negatively charged on the other, which is a characteristic feature of the ADF/cofilin family. The F-loop surface, which is negatively charged on both sides in EhCoactosin, is positively charged on one side and hydrophobic on the other in HCLP (Figure 8B and 8B′).

The surface charge distributions of pfADF1 and pfADF2 also differ from that of EhCoactosin. For pfADF1, one side is highly positively charged and the other has a relatively hydrophobic surface. The N-terminal region of pfADF1 is positively charged relative to that of EhCoactosin [11]. Also, α1 of pfADF1 has three positively charged residues and is relatively long whereas in EhCoactosin it is relatively small and negatively charged [Figure 8C and 8C′]. The surface of pfADF2, while more negatively charged than that of pfADF1, is less negatively charged than that of EhCoactosin (Figure 8D and 8D′). Note that the N-terminal regions of EhCoactosin and PfADF2 were also found to be different; while, as indicated above, the former has Ser repeats, the latter does not [12], [13].

### Comparison of the conformations of EhCoactosin, HCLP, pfADF1, and pfADF2

**Figure 9:**
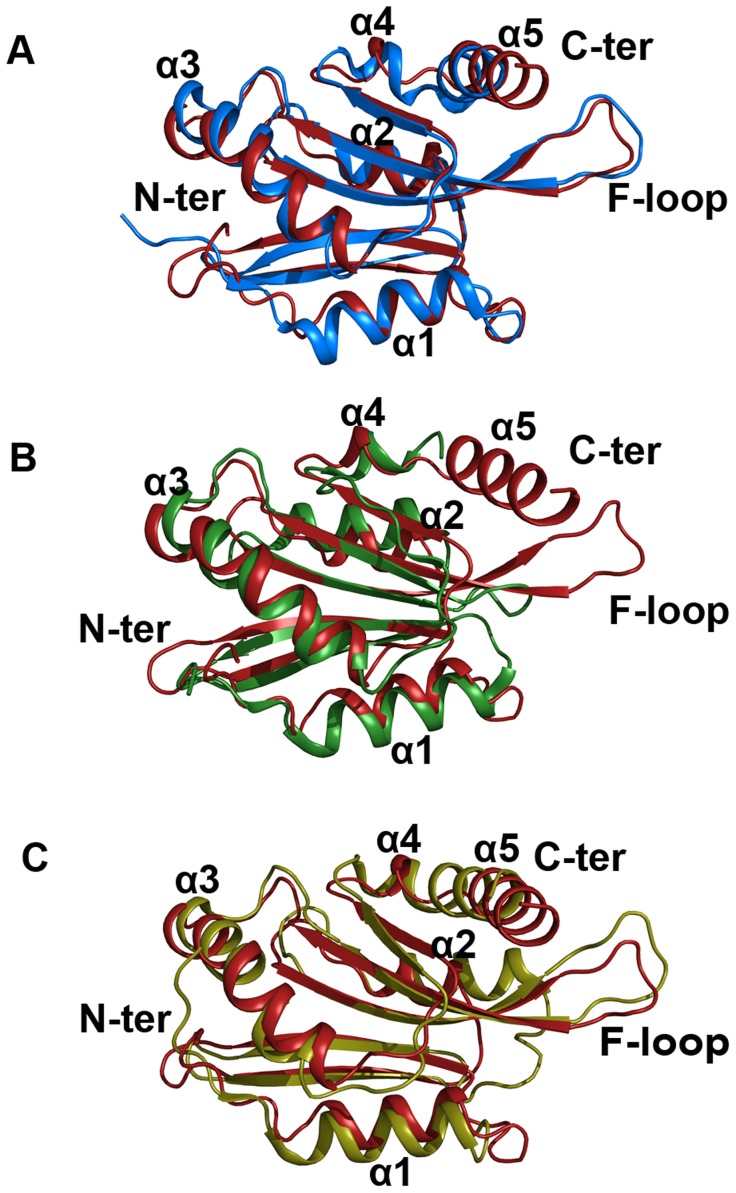
Superimposed images of EhCoactosin (red) with (A) human CLP (blue) RMSD = 1.56 Å, (B) PfADF1 (green) RMSD = 2.0 Å and (C) PfADF2 (yellow) RMSD = 2.13 Å. Superimposition was done using the RAPIDO server, and the figure was prepared with Pymol. In EhCoactosin at the N-terminal (A) end there is no beta sheet but in all these three structures there are beta sheets. When compared with HCLP (A) α1 is shorter in EhCoactosin, but the C-terminal helix of EhCoactosin is longer. In PfADF1 (B) there is no C-terminal helix and F-loop. Its N-terminal α1 is also longer than that of EhCoactosin. In PfADF2 (C) α1 and α2 are longer compared to EhCoactosin. Near the C-terminus, between α4 and α5, there is a loop in EhCoactosin but in PfADF2 it is a continuous stretch of α-helix.

The overall structure of EhCoactosin is quite similar to that of human coactosin-like protein (HCLP), with an RMSD of 1.56 Å and few major differences. The N-terminal regions of the two proteins do deviate by up to 14.7 Å, with that of HCLP bent towards the inside of the structure while in EhCoactosin this N-terminal region is extended. Also, α1 of HCLP is longer by 3 residues compared to that of EhCoactosin (Figure 9A).

The overall structure of EhCoactosin is also fairly similar to the structures of the two types of ADF proteins of *Plasmodium falciparum*, pfADF1 and pfADF2. Although pfADF1 is functionally different than other ADF/Cofilin proteins, since it binds G-actin [12] and only transiently interacts with F-Actin [12], its overall structure differs from that of EhCoactosin by an RMSD of just 2.0 Å. Certain structural differences are quite notable: The F-loop is absent in pfADF1; β3 and β4 of EhCoactosin, which are extended towards its F-loop, are shorter in pfADF1; and a long C-terminal α-helix present in EhCoactosin is absent in pfADF1. All these observations suggest that the F-loop, β3, β4 and the C-terminal helix of EhCoactosin could be involved in binding to F-actin (Figure 9B). Note also that in pfADF1, the N-terminal end is relatively short, and connected to a short β-sheet, which is a characteristic feature of ADF/cofilin, while in EhCoactosin the N-terminal region is long with characteristic serine repeats, which is thought to participate in G-actin binding. However, both these proteins bind G-actin and it is difficult to suggest a possible mechanism with this data.

The RMSD between pfADF2 and EhCoactosin is 2.13 Å. pfADF2 binds F-actin as well as G-actin [12], and in pfADF2, the F-loop, β3, β4, β5 and β6 are similar to those in EhCoactosin. Moreover, the C-terminal helix, which is missing in pfADF1, is present in pfADF2. This helix is nevertheless longer in EhCoactosin. These regions are likely to be involved in F-actin binding (Figure 9C).

### Model of EhCoactosin-actin binding

**Figure 10:**
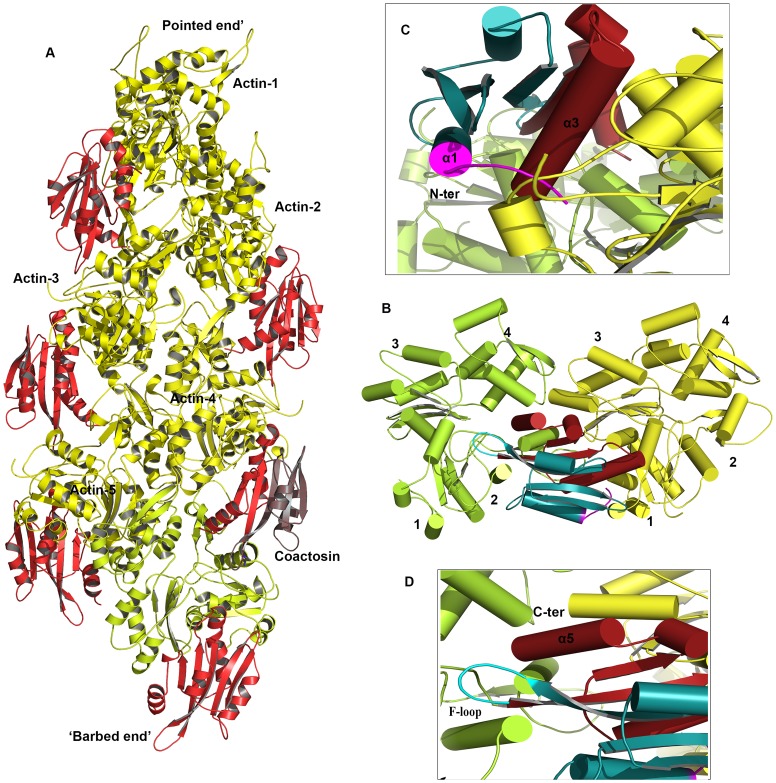
A proposed model of the complex between F-actin and EhCoactosin. Cartoon representations are shown. (A) Actin filament (yellow) bound to EhCoactosin proteins (red), (B) Magnified view of EhCoactosin docked between two actin monomers (green and yellow). The N-terminal half of EhCoactosin (1–75) is shown in teal and the C-terminal half is shown in brick red. (C) A detailed view of the N-terminal binding region (residues 1–7) of EhCoactosin (pink) and the α3 helix interacting with an actin monomer. (D) F-loop and the C-terminal binding region of EhCoactosin interacting with the adjacent actin monomer.

EhCoactosin directly binds F-actin but the mechanism of preventing depolymerisation is not understood. The structural differences of EhCoactosin with Coactosins from other organisms may be responsible for the distinct functional properties. Properties of mutants helped us to model F-actin binding. Here we have sought to analyse the nature of interactions between actin and EhCoactosin by computational modelling. We propose different mode of binding of EhCoactosin to G-actin and F-actin to explain the actin binding properties. Based on the crystal structure of the mouse twinfilin C-terminal ADF homology domain in complex with actin [14] and the recent 9 Å EM model of human Cofilin-2 in complex with actin filaments [15] (Figure S6A and S6B), we built two different models, one for G-actin binding and one for F-actin binding to explain and understand actin binding mechanism of EhCoactosin. EhCoactosin superimposes well with the cofilin of the cofilin-actin complex filaments [15]. In the energy-minimized model, EhCoactosin fits well between the subdomain 1 of the actin monomer and the subdomain 2 of the next actin monomer (Figure 10A). As seen in the model, the N-terminal region of EhCoactosin interacts with subdomain-1 of the actin monomer1 and the C-terminal region of EhCoactosin is placed at the binding interface between two actin molecules (Figure 10B). The α-3 helix forms extensive contacts with subdomain-1 of the actin monomer-1 whereas the F-loop (S69-K75) interacts with the subdomain-2 of the adjacent actin monomer (Figure 10C and D). The C-terminal α-5 helix is docked inside the cavity formed by the two actin molecules. The N-terminal sequence and F-loop region behave like clamps anchoring well within the F-actin structure along the length of the filaments, hence resulting in its stabilization. This explains the effect of EhCoΔF as the mutation of the F-loop results in loss of F-actin stabilization suggesting F-loop is one side of the clamp interacting with F-actin. Thus EhCoΔF can bind F-actin but can't stabilize it. The homology model of the EhCoactosin-F-Actin complex suggests that various regions of the protein, such as the N-terminal sequence, helices α-3 and α-5 and the F-loop play important roles in binding F-actin – and also suggests, in agreement with our mutational studies described above, that no single region or feature of EhCoactosin is indispensible for binding F-actin. Such is the case for EhCoactosin Lys^75^, for example, despite it being conserved and completely responsible for F-actin binding in other systems; EhCoactosin is unique in this regard. EhCoactosin deletion mutants EhCoΔC as well as EhCoΔN also displayed F-actin binding and stabilization abilities similar to that of the wild type protein.

**Figure 11:**
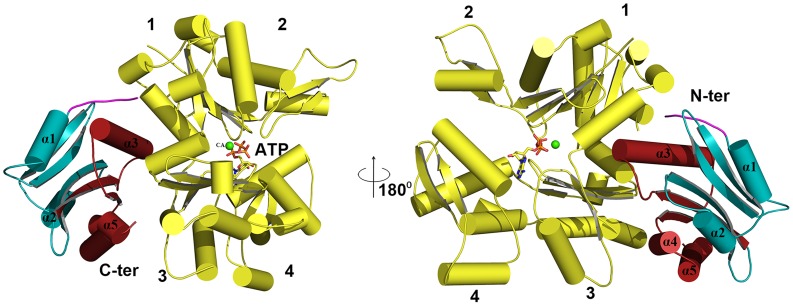
Modelling of G-actin-EhCoactosin complex. An actin molecule with ATP is shown in stick representation and calcium is shown in green. The complex is viewed from opposite sides. The N-terminal half and the C-terminal half of Ehcoactosin are shown in teal and brick red, respectively. The N-terminal loop (residues 1–7) interacting with G-actin is shown in pink. 1,2,3,4 indicate domains of G-actin.

The model for globular monomeric actin (G-actin) binding to EhCoactosin was obtained using the mouse twinflin ADF homology domain in complex with actin (Figure S4B). Based on the energy minimized model, α3 of EhCoactosin binds the cleft between subdomain 1 & 3 of actin as shown in Figure 11. The modelling data suggest that deletion of the N-terminal region and development of positive charge may loosen interaction with a hydrophobic patch on domain 1 of actin (Figure S2). Due to this, α3 can enter in the groove between domain 1 and 3 of G-actin (see G-actin binding model, Figure 11), helping to explain our result described above that EhCoΔN binds G-actin more strongly than does wild-type EhCoactosin. Interestingly the EhCoΔF abolishes G-actin binding suggesting F-loop deletion might have altered the orientation of α3 and thus loss in G-actin binding.

## Discussion

The protist parasite *E. histolytica* undergoes extensive pseudopod extension, and displays high level of motility, phagocytosis and macro-pinocytic activities. These processes are crucial for amebic biology as these are associated with food intake and pathogenesis. Since actin dynamics drives all of these processes, we have been investigating many molecules that are known to participate in actin dynamics. Actin-binding proteins, such as those of the ADF/cofilin family, play a major role in actin dynamics. In the current study, we have investigated structural and functional features of the ADF/cofilin protein EhCoactosin. Our results indicate EhCoactosin to be both a G- and F-actin-binding protein, and that it stabilizes F-actin by direct binding. This set of unusual functional feature is due to presence of unique structural motifs not observed in other coactosins or other homologs.

EhCoactosin displays an overall conformational similarity with other ADF/cofilin family members such as HCLP, pfADF1 and pfADF2, yet also displays distinct differences (Figure S7A). Some of the features, such as presence of helices α1 and α3 at the N-terminal region as well as the F-loop, which contains conserved Lys^75^, are also present in coactosins from other organisms including *D. discoideum* which are structurally conserved in this family (Figure S7B). Distinctive features of EhCoactosin include a longer N-terminal sequence and a more negatively charged surface. As a result of the latter feature, both sides of the F-loop in EhCoactosin is negatively charged while, for example, one side of the F-loop of HCLP is positively charged while the other side is hydrophobic. The observation that certain features found in EhCoactosin are absent in other coactosins suggests that this molecule in *E. histolytica* may impart novel functional properties.

Our data clearly show that EhCoactosin is both an F- and G-actin-binding protein *in vitro*. It is associated with the actin cortex and co-localises with F-actin during pseudopod formation and erythrophagocytosis. The presence of EhCoactosin in phagocytic cups is parallel to the F-actin during the phagocytic cup formation. *In vitro* functional assays suggest that EhCoactosin is a F-actin stabilizing protein which implies its role in maintaining integrity at the leading edge. Nearly all coactosins studied previously, including human CLP, have not shown a direct effect on actin polymerisation or depolymerisation, although they can interfere with capping of filaments. Chick coactosin is an exception which has been shown to be involved in actin polymerisation downstream of Rac signalling and to promote polymerisation [16]. EhCoactosin is a novel member of the coactosin family with direct effect on F-actin stabilization with F-loop playing important role in binding.

The functional difference between EhCoactosin and other coactosins can be attributed mainly to increased length of the N-terminal part and altered charge distribution. These distinct properties of EhCoactosin are likely to contribute to its binding of G-actin and stabilization of F-actin. Deletion of the N-terminal part EhCoactosin, for example, increases the binding affinity for G-actin on the solid phase. The C-terminal part may also have a role in regulating G-actin binding. When it is deleted affinity for G-actin decreases but not drastically and this is similar to HCLP where C-terminal does not play significant role in F-actin binding [17]. Our *in silico* analysis suggests that one molecule of EhCoactosin binds to two adjacent actin molecules in the filament. The binding model also suggests that interactions between EhCoactosin and F-actin involve several regions rather than just the F-loop as in other systems. The N-terminal and F-loop of EhCoactosin function as clamps in F-actin binding and decorate the filament along its length. The long serine rich N-terminal region plays a role in F-actin binding whereas deletion of which results in F-actin severing activity. The model and solid phase data suggest that this may be due to high affinity for G-actin displayed by the mutant as a result of uninhibited binding of α3 between subdomain 1 and 3. Although the Lys^75^ residue is needed by HCLP for binding F-actin, it is not required in case of EhCoactosin since the mutant K75A protein has similar experimentally determined F-actin-binding and other properties as does the wild-type protein. Computational modelling also supports these results as K75A mutant does not show any significant change in binding of EhCoactosin to actin, as K75 is not directly interacting with F-actin. This implies that binding of EhCoactosin and actin involves interactions other than F-loop and Lys^75^ residue unlike other homologs. But complete deletion of F-loop results in loss of F-actin stabilization suggesting F-loop is one side of the clamp interacting with F-actin. Thus EhCoΔF can bind F-actin but can't stabilize it.

Our experiments have shown that EhCoactosin stabilises F-actin, but we also need to understand the underlying contributions to actin dynamics in *E. histolytica* since both depolymerization as well as stability of F-actin are required for critical cellular processes. Many drugs that stabilize F-actin have deleterious effect on processes that require actin dynamics [18], [19], and over-expression of EhCoactosin in *E. histolytica* yields cells that display impaired growth and phagocytosis, presumably due to the protein's stabilization of F-actin. This consequence of overexpression is not seen with other coactosins and appears to be a unique property of the *E. histolytica* protein. *E. histolytica* is an early branching eukaryote displaying unique biology, and although it shares many of the participants of the cytoskeleton remodelling machinery with metazoan organisms, it also uses a few novel proteins in regulating the actin cytoskeleton [1], [2], [3]. The calcium-binding proteins EhCaBP1 and EhCaBP3 are such examples, and they have been shown to be involved in actin dynamics and phagocytic cup formation [2], [3], [20]. All these studies including present study show that *E. histolytica* proteins can also undergo functional diversification in order to fulfil its needs, high rate of actin dynamics. The detailed study of this binding protein will lead to better understanding of the cytoskeletal remodelling in this parasite and also as well evolution of this process in other eukaryotes.

The erythrophagocytosis results indicate *in vitro* concentration of EhCoactosin above critical level may affect actin remodelling. Phagocytosis involves both actin polymerisation and depolymerisation which is mediated by several actin-binding proteins. The high levels of EhCoactosin in cell may promote excess stability of F-actin *in vitro* by preventing access of actin remodelling protein to F-actin required during the phagocytosis. Taken together this leads to increased rigidity in actin cytoskeleton which impairs its dynamic remodelling required for processes like motility and phagocytosis.

In conclusion, EhCoactosin is directly involved in F-actin stabilization, which has not been reported earlier. *In vivo* EhCoactosin may actively contribute to the maintenance of F-actin during erythrophagocytosis and pseudopod formation. The interactions between EhCoactosin and F-actin depend on several regions in the protein rather than specific residues such as Lys^75^. The evolutionary basis of development of specific interaction in higher organisms can be understood by studying primitive eukaryotes like *E. histolytica*. This study will also lead to better understanding of actin dynamics in this organism and as well as evolution of actin dynamics as a process in organisms.

## Materials and Methods

### Cloning of various constructs

The coding sequence of coactosin gene (GenBank accession no. XP_650926) was amplified by PCR from genomic DNA of *Entamoeba histolytica* strain HM1:IMSS using the forward primer 5′-CCGCCATGGCAATGTCTGGATTTGATCTTAG-3′ and the reverse primer 5′-CCGCTCGAGCTTAATTTTAGCAGCGATTTC-3′. The EhCoactosin gene was cloned in pET28b (Novagen) between Nco1 and Xho1 sites with a C-terminal 6× His tag. Four constructs were prepared for biochemical experiments: wild-type EhCoactosin (EhCoWT); an EhCoactosin in which 14 amino acid residues were deleted from the C-terminus because it was predicted to form a loop (EhCoΔC); another for which 7 residues were deleted from the N terminus (EhCoΔN), F-loop spanning from 71–76 amino acid was also deleted (EhCoΔF) and a single site substitution mutant (K75A). The cloning was confirmed by restriction digestion by Nco1 and Xho1 followed by DNA sequencing. The CAT gene of the shuttle vector pEhHYG-tetR-O-CAT (TOC) was excised using KpnI and BamHI and the EhCoactosin gene was inserted in its place in either the sense or the antisense orientation. The expression in this vector was tetracycline inducible and expressed sense (S) and antisense (AS) RNA of the gene in *E. histolytica* trophozoites. For the study of co-localization in *E. histolytica* cells we carried out HA tagging at the N-terminus of EhCoactosin. The Forward Primer **5′**-CGGGGTACCATGTATCC ATATGATGTTC CAGATTATGCTATGTCTGGATTTG-**3′** and the reverse primer **5′**- GCGGGATCCTTAAGCATAATCTGGAACATCATATGGATAATT TGAGGTGG-**3′** were used for HA tagging.

### Over-expression and purification of the various proteins

The recombinant plasmid containing the EhCoactosin gene was transformed into *E. coli* BL21 (DE3) cells (Novagen). Primary culture was grown overnight in 50 ml LB media from the single colony of transformed BL21 cells supplemented with 50 µg/ml Kanamycin at 37°C. Secondary culture was grown by inoculating 1% of primary culture in the same media at 37°C until the OD_600_ reached 1.0. The culture was induced with 1 mM isopropyl β-D-1-thiogalactopyranoside (IPTG) (Sigma) and allowed to grow for another 4 hrs at the same temperature. Cells were harvested by centrifugation at 6000 rpm for 10 minutes at 4°C. These cells were stored at −80°C until further processing.

The harvested cells were resuspended and homogenized in resuspension buffer containing 50 mM Tris HCl (pH 8.0), 0.1 mM EDTA and 0.1 mM DTT. Resuspended cells were lysed with 3 cycles of flash-freezing in liquid nitrogen and subsequent thawing in water-bath at 37°C. The lysate was subjected to 5–6 cycles of sonication on ice at 25% amplitude with each pulse of 30 sec and 1 min interval. The sonicated cell lysate was centrifuged at 13,000 rpm for 30 minutes at 4°C. Supernatant was filtered with Whatman filter paper no. 1 and clear lysate was passed through a Nickel-NTA column (GE healthcare) pre-equilibrated with resuspension buffer. Thereafter, the column was washed with 2 bed volumes of buffer containing 50 mM Tris HCl (pH 8.0), 0.1 mM EDTA, 0.1 mM DTT and 10 mM imidazole. The bound protein was eluted with buffer comprising 50 mM Tris-HCl (pH 8.0), 0.1 mM EDTA, 0.1 mM DTT and 100 mM imidazole. The purified fractions of protein were concentrated using Centricon filters (Millipore) and subjected to gel filtration chromatography on HiLoad Superdex 75G 16/60 column (GE Healthcare) pre-equilibrated with buffer containing 50 mM Tris-HCl (pH 8.0), 0.5 mM EDTA, 0.5 mM DTT and 1 mM sodium azide. Homogeneity of protein was assessed on 12% SDS-PAGE (Figure S8). Peak fractions were concentrated using Centricon filters (Millipore) and concentration was estimated with A_280_.

Selenomethionine-labelled EhCoactosin was purified under reducing conditions using specific media (by Molecular Dimensions, United Kingdom). The concentration of selenomethionine was maintained at about 25 mg/litre. Initially, the primary culture was grown in LB medium overnight. Cells were then harvested by centrifuging at 4000 rpm for 6 min. Harvested cells were resuspended in the complete selenomethionine media, and washed once with same media to completely remove any leftover LB medium. Secondary culture was grown by inoculating 1% of primary culture in the same media at 37°C until the OD_600_ reached 1.0. Culture was allowed to grow at 37°C for about 4 hrs after inoculation until OD_600_ reached 1.0. Cells were induced with 1 mM IPTG and allowed to grow for another 4 hrs at same temperature. Cells were harvested at 6,500 rpm for 6 min and stored at −80°C for further processing. Subsequent processing and purification were done by the same method used for native EhCoactosin.

G-actin was purified from rabbit skeletal muscle acetone powder [21]. Further Actin was labelled with N-(1-pyrene) iodoacetamide (P-29, Molecular Probes) by the protocol described previously [22] for performing the pyrene-actin assay.

### Crystallization and data collection

Native EhCoactosin protein was crystallized using the hanging drop vapor diffusion method in 24-well Linbro plates against a reservoir solution containing 25–35% PEG 1500, 100 mM sodium acetate, 0.2 mM CaCl_2_, 10 mM MgCl_2_ and 100 mM HEPES, pH 7.3–7.7. Two µl of [∼75 mg/ml] protein and 2 µl of reservoir solution were mixed and allowed to equilibrate at 16°C. The crystals that formed in these drops were flash frozen in a cryoprotectant solution containing additional 5% PEG 400 mixed with mother liquor. Selenomethionine-labelled protein was prepared and crystallized using similar conditions. The crystal appeared in condition containing 28–33% PEG 3350, 100 mM sodium acetate, 0.2 mM CaCl_2_, 10 mM MgCl_2_, 5% isopropanol and 100 mM HEPES pH 7.4–7.7 (Figure S9). The crystals were flash frozen in the same cryo-protectant. The X-ray data for selenomethionine-substituted crystals were collected at the BM14 synchrotron beamline, ESRF, Grenoble, France at a selenium peak wavelength of 0.97860 Å. Data sets were indexed and scaled using HKL2000 [23].

### Structure solution and refinement

Anomalous data collected for Se-Met labelled EhCoactosin crystals were used to calculate FA values using the program SHELXC [24]. Each of the two heavy atoms expected were found using the program SHELXD [24]. Initial phases were calculated after density modification using SHELXE [25]. The reflection file was further used in the Autobuild program [25] of the Phenix suite [26] for automated model building. Then missing residues were traced into the electron density and refined by iterative model building using the COOT graphics package combined with REFMAC5 [27]. HEPES, Na, and water molecules were added by COOT guided by Fo-Fc electron density >3σ. The final model was validated by the Procheck [28] program of the CCP4 suite. Structure factors and co-ordinates have been validated and deposited in the Protein Data Bank with accession id 4LIZ. Data statistics are listed in Table 1.

**Table 1:** Data collection and refinement statistics.

Space group	P6_5_
Wavelength [Å]	0.97372
Unit Cell Parameters [Å] a,b,c	76.6, 76.6, 54.6
α, β, γ [°]	90, 90, 120
Resolution [Å]	1.49
Resolution range	42.21-1.49 [1.49–1.54]^a^
Completeness [%]	98.1 [82.3]^a^
R_merge_ [%]	5.4 [33.1]^a^
{I/σ[I]}	57.27 [2.97]^a^
Multiplicity	8.6 [3.5]^a^
Mosaicity	0.6
**Refinement Statistics**	
R factor	16.2 [27.0]^a^
Free_R	18.8 [28.9]^a^
B factor	26.3
**rmsd**	
Bond Angle [°]	2.68
Bond Length [Å]	0.027
**Ramachandran Statistics**	
Favored [%]	99.3
Allowed [%]	0.7
Disallowed [%]	0
ESU Based on Free_R	0.064

a Numbers in parentheses are for the last resolution shell.

### Modelling of EhCoactosin complexes

A model of the F-actin–EhCoactosin complex was built using the 9 Å electron microscopy derived model of F-actin ADF/cofilin protein complex [16]. The crystal structure of EhCoactosin was then superimposed onto the human ADF/cofilin molecule from the EM model using the RAPIDO server [29]. The ADF/cofilin molecule used showed an extended N-terminal region which did not superimpose well. The overall structure (119 atoms), however, did superimpose well with an RMSD of 1.15 Å (Figure S4A). The final model of the complex with five actin and six coactosin molecules was then subjected to energy minimization with 2500 cycles of steepest descent and followed by 2500 cycles of steepest descent algorithm using AMBER molecular dynamics package [30]. Similarly the model of the G-actin-EhCoactosin complex was obtained using the crystal structure of mouse twinfilin C-terminal ADF homology domain in complex with actin [14]. The root mean square deviation obtained was 2.17 Å (Figure S4B). The electrostatic surface charge distribution was calculated using the ABPS plugin in PyMOL. The negative electrostatic surface is shown in red, and the positive surface in shown in blue; all surfaces are drawn at 3 e/kBT. The images were prepared using Pymol software [31].

### Growth and maintenance of parasite *E. histolytica* strain HM1-IMSS

*E. histolytica* strain HM-1: IMSS and all transformed parasites were maintained and grown in TYI-S-33 medium [1] containing 125 ml of 250 U ml^−1^ benzyl penicillin and 0.25 mg ml^−1^ streptomycin per 100 ml of medium. The transformants containing tetracycline inducible system were grown in presence of 10 µg ml^−1^ of Hygromycin B. The cells were first grown for 48 h (60–70% confluent) and then 20 µg ml^−1^ tetracycline was added to the medium for 36 h for induction. Cells carrying constructs with constitutive expression system (such as GFP) were maintained at 10 µg ml^−1^ of G418. But the experiments were carried out in presence of 30 µg ml^−1^ of G418.

### Transfection and selection of *E. histolytica* trophozoites

Transfection was performed by electroporation. Briefly, trophozoites in log phase were harvested and washed with phosphate buffer saline (PBS), followed by incomplete cytomix buffer (10 mM K_2_HPO_4_/KH_2_PO_4_ (pH 7.6), 120 mM KCl, 0.15 mM CaCl_2_, 25 mM HEPES (pH 7.4), 2 mM EGTA, 5 mM MgCl_2_]. The washed cells were then re-suspended in 0.8 ml of complete cytomix buffer (incomplete cytomix containing 4 mM adenosine triphosphate, 10 mM glutathione) containing 200 mg of plasmid DNA and subjected to two consecutive pulses of 3000 V/cm (1.2 kV) at 25 mF (Bio-Rad, electroporator). The transfectants were initially allowed to grow without any selection. Drug selection was initiated after 2 days of transfection in the presence of 10 µg ml^−1^ G-418 for constructs with GFP or 10 µg ml^−1^ of hygromycin B was used for tetracycline inducible constructs.

### Immunofluorescence staining

Immunofluorescence staining was carried out as described previously [1]. Briefly *E. histolytica* cells resuspended in TYI-33 medium were transferred onto acetone-cleaned coverslips placed in a petri dish and allowed to adhere for 10 min at 35.5°C. The culture medium was removed and cells were fixed with 3.7% pre-warmed paraformaldehyde (PFA) for 30 min. After fixation, the cells were permeabilized with 0.1% Triton X-100/PBS for 1 min. This step was omitted for non-permeabilized cells. The fixed cells were then washed with PBS and quenched for 30 min in PBS containing 50 mM NH_4_Cl. The coverslips were blocked with 1% BSA/PBS for 30 min, followed by incubation with primary antibody at 37°C for 1 h. The cover slips were washed with PBS followed by 1% BSA/PBS before incubation with secondary antibody of 30 min at 37°C. Antibody dilutions used were: Anti- EhCoactosin at 1∶200, anti-HA at 1∶50, TRITC-Phalloidin at 1∶250 and anti-rabbit Alexa 488 (Molecular Probes) at 1∶300. The preparations were further washed with PBS and mounted on a glass slide using DABCO (1,4-diazbicyclo [2,2,2] octane (Sigma) 10 mg/ml in 80% glycerol). The edges of the coverslip were sealed with nail-paint to avoid drying. Confocal images were visualized using an Olympus FluoView FV1000 laser scanning microscope.

### Actin filament de-polymerization assay

17.5 µM G-actin with 10% pyrene labeled was polymerized for one and half hour at 25°C in F-buffer (10 mM Tris-Cl pH8.0, 0.2 mM DTT, 0.7 mM ATP, 50 mM KCl, 2 mM MgCl_2_). Depolymerization kinetics was started with the addition of 2 µL of preassembled actin with 58 µL of F-buffer, and the volume was made up to 70 µl with HEKG5 or protein solution. N-pyrene fluorescence was monitored with excitation at 365 nm and emission was measured at 407 nM for 600 seconds (QM 40 PTI NJ). The de-polymerizing protein Xenopus cofilin1 (Xac1) was used as a positive control [10].

### Solid-phase assay

The solid-phase assay experiments were performed to monitor the binding of wt- and mutant EhCoactosin proteins to G-actin. The wells of the ELISA plate were coated with 5 µM G-actin in PBS buffer and incubated for 12 h at 4°C. The wells were washed with PBS-T buffer. 5 µM protein was added to the wells in duplicates. Bound protein was detected with anti-EhCoactosin antibody followed by HRPO-lined anti-rabbit IgG using the colorimetric substrate TMB (Sigma). The reaction was stopped with 2N H_2_SO_4_ and absorbance was monitored at 405 nm with ELISA plate reader (Bio-Rad, USA).

### Co-sedimentation assay

5 µM of rabbit muscle actin was polymerized for one and half hour at 25°C in F-buffer (10 mM Tris-Cl pH8.0, 0.2 mM DTT, 0.7 mM ATP, 50 mM KCl, 2 mM MgCl_2_). After polymerization, actin was mixed with appropriate target protein (5 µM) in a total volume of 150 µl and incubated for 30 min at RT. The samples were centrifuged at 100,000 g for 45 min at 4°C. The supernatant and pellet fractions (total) were analyzed by 12% SDS-PAGE followed by Coomassie blue staining. All target proteins were ultracentrifuged at 1,00,000× g for 1 h and the supernatant was used for the assays in order to avoid aggregates.

### Erythrophagocytosis assay

The human erythrocytes used in the experiments were collected from Somlata. The blood was taken by piercing the ring finger by sterile needle and transferred into a sterile tube containing PBS. 10^7^ red blood cells (RBC) were washed with PBS and incomplete TYI-33 and were incubated with 10^5^ amoeba for varying time periods at 37°C in 0.5 ml culture medium. The amoebae and erythrocytes were pelleted down, non-engulfed RBCs were bursted with cold distilled water and recentrifuged at 1000 g for 2 min. This step was repeated twice, followed by resuspension in 1 ml formic acid to burst amoebae containing engulfed RBCs. The absorbance was measured at 400 nm.

### Ethics statement

The human erythrocytes used in the experiments were collected from Somlata. The blood was taken by piercing the ring finger by sterile needle and transferred into a sterile tube containing PBS. The consent letter was obtained from the individual for taking blood sample before carrying out the experimental studies.

## Supporting Information

Figure S1 G-actin binding assay with (A) EhCoΔN and (B) EhCoΔC where both the protein showed effect in dose dependent manner. (C) In Solid phase assay, WT and mutant proteins were incubated with G-actin-coated wells of a multi-well plate as described in the text. Binding was carried out either in the presence of Ca^2+^ (2 mM) or EGTA (5 mM), as indicated. EhCaBP1 binding was quantified by ELISA using an antibody against EhCoactosin. EhCaBP1 served as positive control while EhCaBP2 served as negative control for the assay.
(TIF)

Figure S2 Comparison of surface charge distribution of (A) EhCoactosin and (B) N-terminus-deleted EhCoactosin. The N-terminal region of wild-type EhCoactosin has a hydrophobic surface but after deleting seven amino acids from this end there is cluster of positive charges that get formed and exposed at this end. This deletion makes the N-terminal half of the protein positively charged overall compared to the C-terminal half which is highly negatively charged.
(TIF)

Figure S3 GST-bead pull down assay to determine direct interaction between Xac1 and EhCoactosin. The GST-tagged Xac1 was used and incubated with the mentioned proteins along with control GST tag alone. The blot was probed by anti-EhCoactosin antibody and GST antibody to determine the pulled down proteins.
(TIF)

Figure S4 (A) Expression of HA-tagged EhCoactosin in trophozoites. The trophozoites were treated with 30 µg/mL of G418 for 48 h. The expression was detected by western blotting using anti-HA antibody. For confirming equal loading, endogenous protein EhCaBP1 was probed by specific antibody which remained unaffected during induction. (B) Localisation of HA-tagged protein in trophozoites.
(TIF)

Figure S5 (A) Schematic representation of constructs used for expressing sense RNA and antisense RNA in *E. histolytica* trophozoites. Restriction digestion by Kpn1 and BamH1 of (B) sense construct (C) and antisense construct.
(TIF)

Figure S6 Structural superimposition of EhCoactosin on A) the human ADF/cofilin molecule derived from EM (see main text) and on B) the crystal structure of mouse twinfilin C-terminal ADF homology domain.
(TIF)

Figure S7 (A) Structural superimposition of EhCoactosin (brick red) with HCLP(blue), PfADF1(green) and PfADF2 (yellow) (See Figure 9 in main text for separate superpositions). (B) Structure based sequence alignment of EhCoatosin, HCLP, PfADF1, PfADF2. The helices and β strands are shown at the top. The sequence alignment was performed using clustalX and the image was prepared using ESPript server.
(TIF)

Figure S8 (A) SDS PAGE showing EhCoactosin purified by Ni-NTA affinity chromatography. (B) SDS PAGE showing further purification of affinity purified protein by gel exclusion chromatography. (C) Gel exclusion chromatography elution profile of EhCoactosin, which indicates the pure recombinant protein.
(TIF)

Figure S9 Crystal of EhCoactosin (Selenomethionine incorporated).
(TIF)

Text S1 PDB Validation report of the structural coordinates 4LIZ.
(PDF)
