# Supplementary figures and images for: EhCoactosin Stabilizes Actin Filaments in the Protist Parasite Entamoeba histolytica
Source: PLoS Pathog. 2014 Sep 11;10(9):e1004362. doi: 10.1371/journal.ppat.1004362 (PMC4161475; doi:10.1371/journal.ppat.1004362)

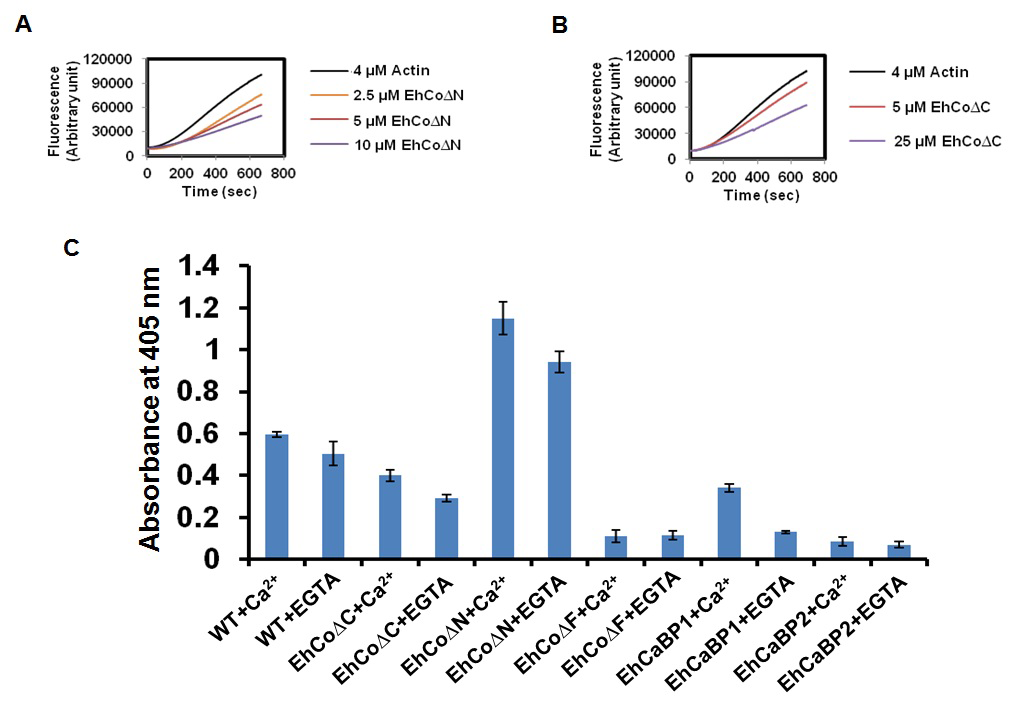

Supplement: Figure S1 — G-actin binding assay with (A) EhCoΔN and (B) EhCoΔC where both the protein showed effect in dose dependent manner. (C) In Solid phase assay, WT and mutant proteins were incubated with G-actin-coated wells of a multi-well plate as described in the text. Binding was carried out either in the presence of Ca2+ (2 mM) or EGTA (5 mM), as indicated. EhCaBP1 binding was quantified by ELISA using an antibody against EhCoactosin. EhCaBP1 served as positive control while EhCaBP2 served as negative control for the assay. (TIF) [file ppat.1004362.s001.tif]

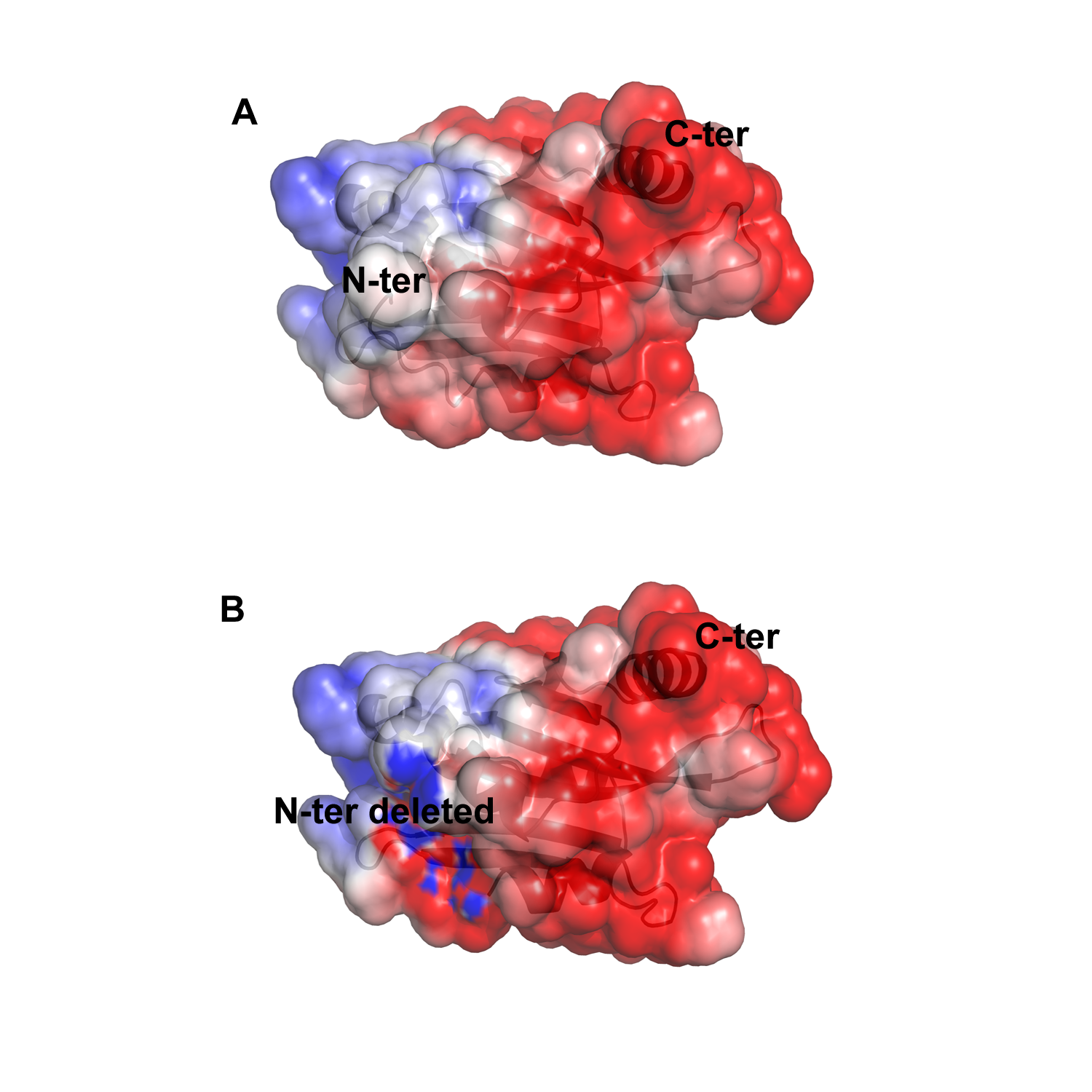

Supplement: Figure S2 — Comparison of surface charge distribution of (A) EhCoactosin and (B) N-terminus-deleted EhCoactosin. The N-terminal region of wild-type EhCoactosin has a hydrophobic surface but after deleting seven amino acids from this end there is cluster of positive charges that get formed and exposed at this end. This deletion makes the N-terminal half of the protein positively charged overall compared to the C-terminal half which is highly negatively charged. (TIF) [file ppat.1004362.s002.tif]

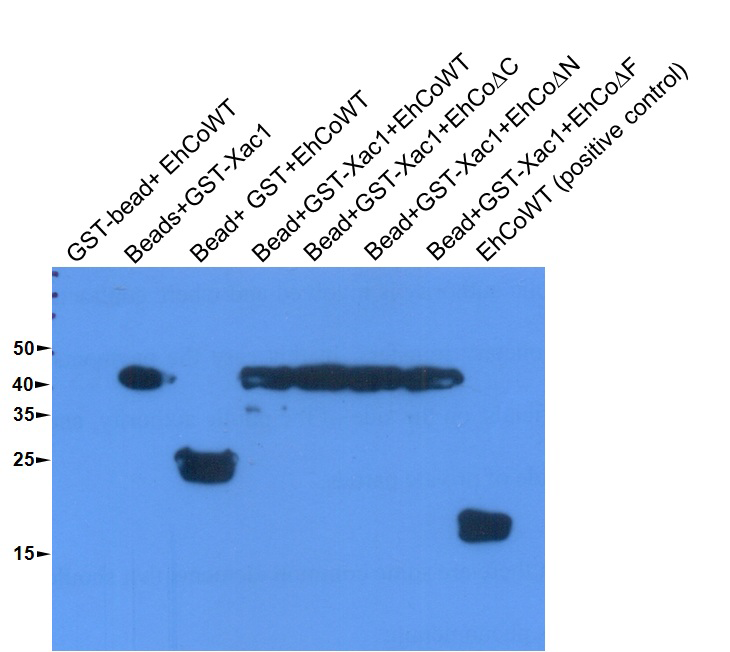

Supplement: Figure S3 — GST-bead pull down assay to determine direct interaction between Xac1 and EhCoactosin. The GST-tagged Xac1 was used and incubated with the mentioned proteins along with control GST tag alone. The blot was probed by anti-EhCoactosin antibody and GST antibody to determine the pulled down proteins. (TIF) [file ppat.1004362.s003.tif]

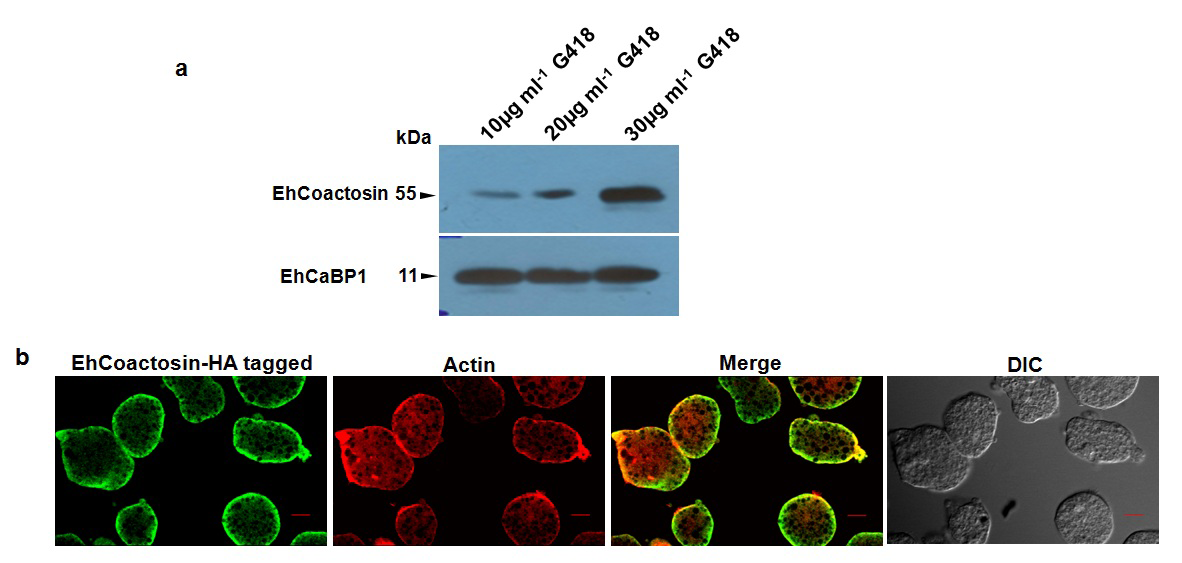

Supplement: Figure S4 — (A) Expression of HA-tagged EhCoactosin in trophozoites. The trophozoites were treated with 30 µg/mL of G418 for 48 h. The expression was detected by western blotting using anti-HA antibody. For confirming equal loading, endogenous protein EhCaBP1 was probed by specific antibody which remained unaffected during induction. (B) Localisation of HA-tagged protein in trophozoites. (TIF) [file ppat.1004362.s004.tif]

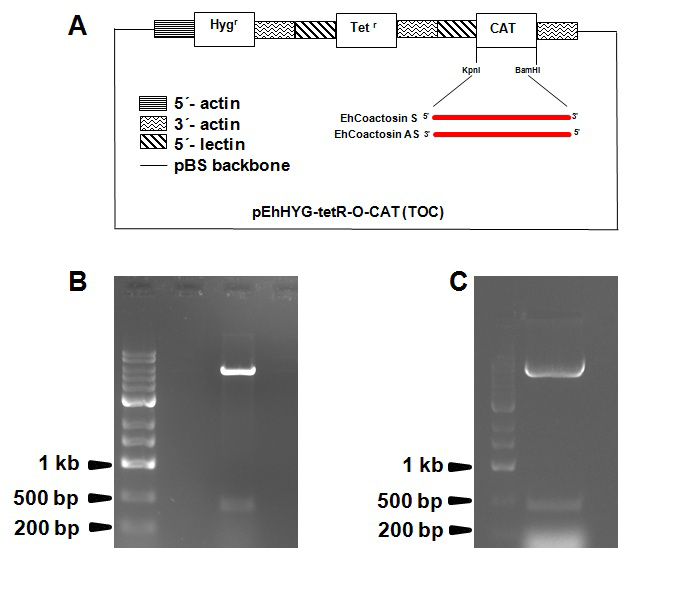

Supplement: Figure S5 — (A) Schematic representation of constructs used for expressing sense RNA and antisense RNA in E. histolytica trophozoites. Restriction digestion by Kpn1 and BamH1 of (B) sense construct (C) and antisense construct. (TIF) [file ppat.1004362.s005.tif]

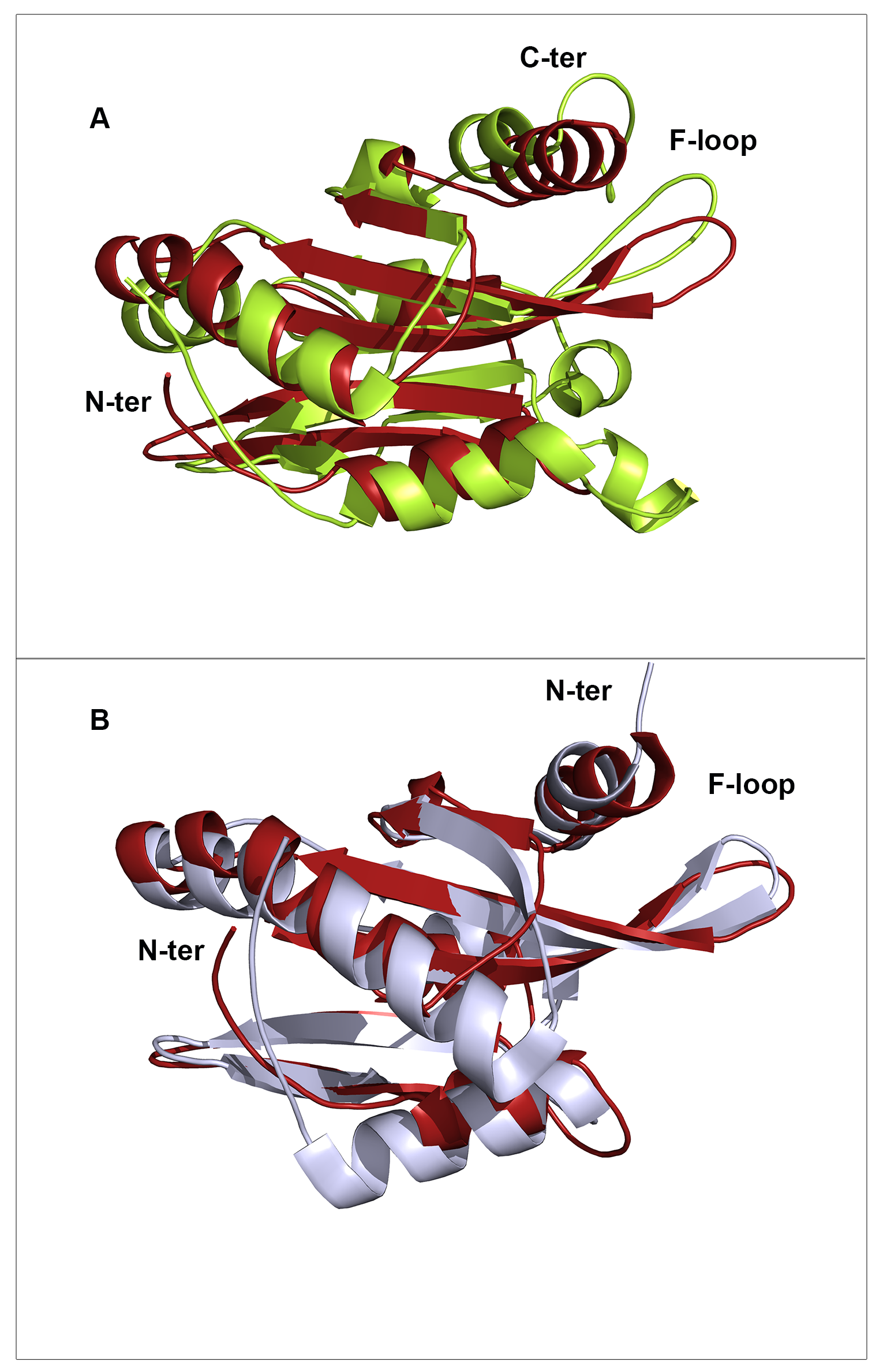

Supplement: Figure S6 — Structural superimposition of EhCoactosin on A) the human ADF/cofilin molecule derived from EM (see main text) and on B) the crystal structure of mouse twinfilin C-terminal ADF homology domain. (TIF) [file ppat.1004362.s006.tif]

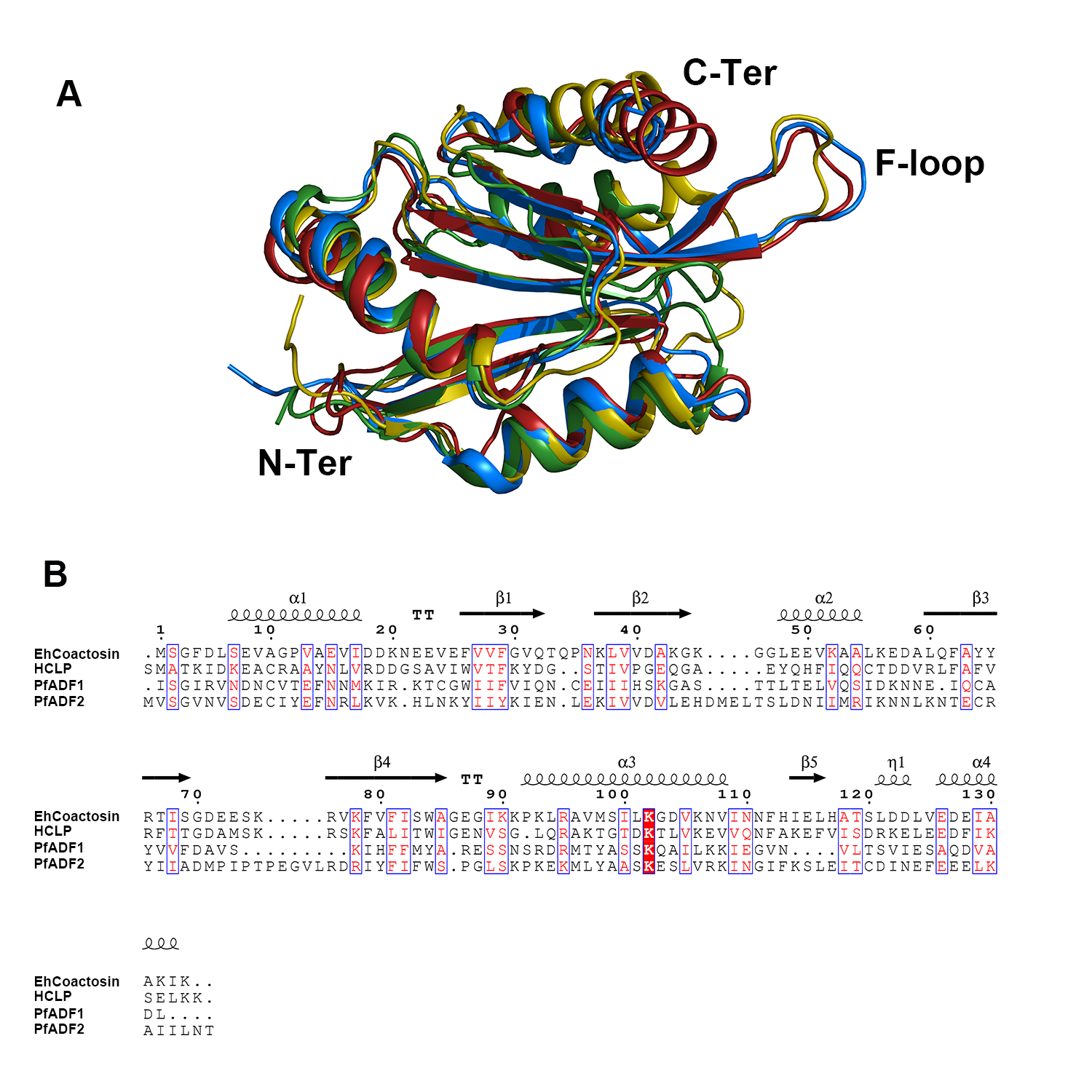

Supplement: Figure S7 — (A) Structural superimposition of EhCoactosin (brick red) with HCLP(blue), PfADF1(green) and PfADF2 (yellow) (See Figure 9 in main text for separate superpositions). (B) Structure based sequence alignment of EhCoatosin, HCLP, PfADF1, PfADF2. The helices and β strands are shown at the top. The sequence alignment was performed using clustalX and the image was prepared using ESPript server. (TIF) [file ppat.1004362.s007.tif]

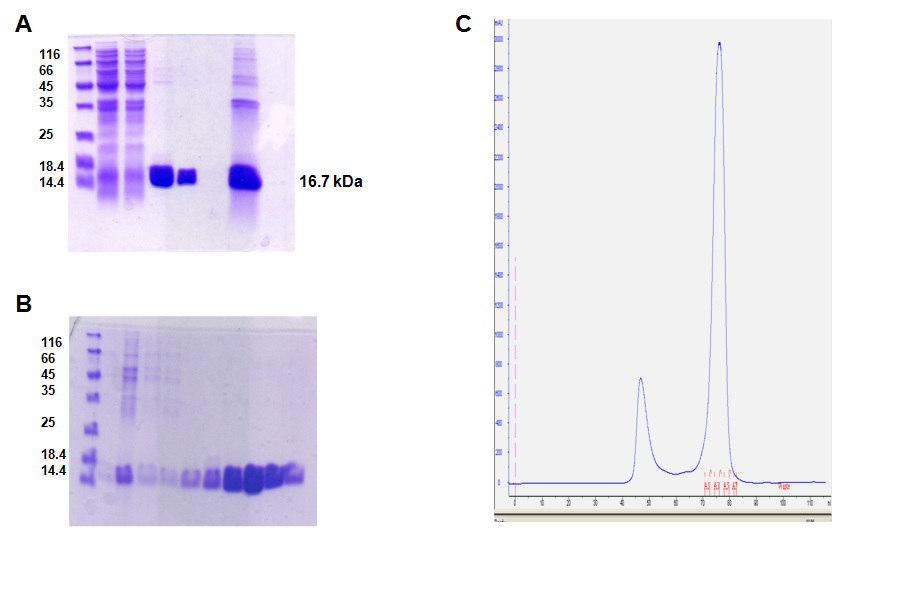

Supplement: Figure S8 — (A) SDS PAGE showing EhCoactosin purified by Ni-NTA affinity chromatography. (B) SDS PAGE showing further purification of affinity purified protein by gel exclusion chromatography. (C) Gel exclusion chromatography elution profile of EhCoactosin, which indicates the pure recombinant protein. (TIF) [file ppat.1004362.s008.tif]

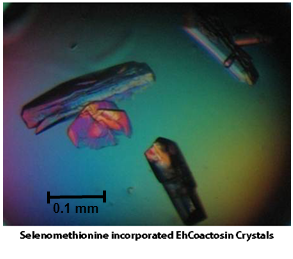

Supplement: Figure S9 — Crystal of EhCoactosin (Selenomethionine incorporated). (TIF) [file ppat.1004362.s009.tif]
