# Supplementary material for: EhCoactosin Stabilizes Actin Filaments in the Protist Parasite Entamoeba histolytica
Source: PLoS Pathog. 2014 Sep 11;10(9):e1004362. doi: 10.1371/journal.ppat.1004362 (PMC4161475; doi:10.1371/journal.ppat.1004362)
Supplement: Text S1 — PDB Validation report of the structural coordinates 4LIZ. (PDF) [file ppat.1004362.s010.pdf]

PDB ID : 4LIZ
RCSB ID : RCSB080688
TITLE : Crystal structure of coactosin from Entamoeba histolytica
AUTHORS : S.Gourinath, N.Kumar

The following geometrical and stereochemical features have been calculated for your structure.

CLOSE CONTACTS

==> Close contacts in same asymmetric unit. Distances smaller than 2.2 Angstroms are considered as close contacts.

Chain	Atom	Res	Seq		Chain	Atom	Res	Seq	Symm_Code	Distance
A	SE	SE	203	-	A	SE	SE	205	(1, 5, 5, 5)	Dist = 1.20
A	O	HOH	467	-	A	O	HOH	510	(1, 5, 5, 5)	Dist = 2.05
A	O	HOH	368	-	A	O	HOH	402	(1, 5, 5, 5)	Dist = 2.09
A	O	HOH	378	-	A	O	HOH	406	(1, 5, 5, 5)	Dist = 2.14
A	O	HOH	415	-	A	O	HOH	506	(1, 5, 5, 5)	Dist = 2.16

==> Close contacts based on crystal symmetry. Distances smaller than 2.2 Angstroms are considered as close contacts.

Chain	Atom	Res	Seq		Chain	Atom	Res	Seq	Symm_Code	Distance
A	SE	SE	202	-	A	SE	SE	201	(5, 5, 4, 4)	Dist = 2.19

BOND DISTANCES AND ANGLES

Bond and angle checks are performed by first computing the average rms error for all bonds and angles relative to standard values for nucleotide units [L. Clowney et al., Geometric Parameters in Nucleic Acids: Nitrogenous Bases, J.Am.Chem.Soc. 1996, 118, 509-518; A. Gelbin et al., Geometric Parameters in Nucleic Acids: Sugar and Phosphate Constituents, J.Am.Chem.Soc. 1996, 118, 519-529] and amino acid units [R.A. Engh and R. Huber, Structure quality and target parameters, International Tables for Crystallography, Volume F, 2001, 382-392]. Any bond or angle which deviates from the dictionary values by more than six times this computed rms error is identified as an outlier.

==> Covalent Bond Lengths:

The overall RMS deviation for covalent bonds relative to the standard dictionary is 0.028 Angstroms

The following table contains a list of the covalent bonds greater than 6 times standard deviation.

Deviation	Residue Name	Chain ID	Sequence Number	AT1	-	AT2	Bond Distance	Dictionary Value	Standard Deviation
-0.110	SER	A	120	CB	-	OG	1.308	1.418	0.013

1 out of total 1097 covalent bonds (0.091%) have greater than 6 times standard deviation.

==> Covalent Angle Values:

The overall RMS deviation for covalent angles relative to the standard dictionary is 2.6 degrees.

The following table contains a list of the covalent bond angles greater than 6 times standard deviation.

Deviation	Residue Name	Chain ID	Sequence Number	AT1	-	AT2	-	AT3	Bond Angle	Dictionary Value	Standard Deviation
-10.0	ASP	A	18	CB	-	CG	-	OD2	108.3	118.3	0.9
7.9	ASP	A	41	CB	-	CG	-	OD1	126.2	118.3	0.9
-11.6	GLU	A	50	OE1	-	CD	-	OE2	111.7	123.3	1.2
4.8	ARG	A	66	NE	-	CZ	-	NH1	125.1	120.3	0.5
-6.5	ARG	A	66	NE	-	CZ	-	NH2	113.8	120.3	0.5
-6.5	ASP	A	123	CB	-	CG	-	OD1	111.8	118.3	0.9
6.9	ASP	A	127	CB	-	CG	-	OD1	125.2	118.3	0.9
-7.5	ASP	A	127	CB	-	CG	-	OD2	110.8	118.3	0.9

8 out of total 1473 bond angles (0.543%) have greater than 6 times standard deviation.

TORSION ANGLES

The torsion angle distributions have been checked. To view these reports, please refer to the ADIT Validation Server at http://deposit.pdb.org/validate.

==> The following table contains a list of torsion angles outside the expected Ramachandran regions [GJ. Kleywegt and TA. Jones, PHI/PSI-chology: Ramachandran Revisited, Structure 1996, 4, 1395 - 1400].

Residue	Chain	Sequence	PSI	PHI
GLU	A	72	-75.15	-86.20

==> The following residues have unexpected configuration of the chiral center using CA--C--CB--N chirality for amino acids and C1'--O4'--N1(N9)--C2' chirality for nucleic acids.

Residue	Chain	Sequence	Improper	Details
SER	A	2	24.64	Expecting L Found L OUTSIDE RANGE

CHIRALITY

The chirality has been checked. O1P, O2P, and hydrogen atoms which do not follow the convention defined in the IUBMB (Liebecq, C. Compendium of Biochemical Nomenclature and Related Documents, 2nd ed.; Portland Press: London and Chapel Hill, 1992) and IUPAC nomenclature (J.L. Markley, A. Bax, Y. Arata, C.W. Hilbers, R. Kaptein, B.D. Sykes, P.E. Wright and K. Wuthrich, Recommendations for the Presentation of NMR Structures of Proteins and Nucleic Acids, Pure & Appl. Chem., Vol. 70, pp. 117-142, 1998) have been standardized. Any other stereochemical violations are listed below.

none

SOLVENT

The following solvent molecules are further than 3.5 Angstroms away from macromolecule atoms in the asymmetric unit that are available for hydrogen bonding. Solvent molecules in extended hydration shells separated by 3.5 Angstroms or less are not listed.

none

We have replaced the coordinates for solvent molecules which could be translated back into the asymmetric unit. Please review all solvent molecules in your file and contact us if you have any serious objections.

MISSING RESIDUES

```
==> The following residues are missing:
    (Note: The SEQ number starts from 1 for each chain according to SEQRES
     sequence record.)

     RES MOD#C SEQ

     HIS(    A 140 )
     HIS(    A 141 )
     HIS(    A 142 )


   PDB Chain_ID: A

           1                                                           15
   SEQRES: MET SER GLY PHE ASP LEU SER GLU VAL ALA GLY PRO VAL ALA GLU
   COORDS: MET SER GLY PHE ASP LEU SER GLU VAL ALA GLY PRO VAL ALA GLU
           1                                                           15

           16                                                          30
   SEQRES: VAL ILE ASP ASP LYS ASN GLU GLU VAL GLU PHE VAL VAL PHE GLY
   COORDS: VAL ILE ASP ASP LYS ASN GLU GLU VAL GLU PHE VAL VAL PHE GLY
           16                                                          30

           31                                                          45
   SEQRES: VAL GLN THR GLN PRO ASN LYS LEU VAL VAL ASP ALA LYS GLY LYS
   COORDS: VAL GLN THR GLN PRO ASN LYS LEU VAL VAL ASP ALA LYS GLY LYS
           31                                                          45

           46                                                          60
   SEQRES: GLY GLY LEU GLU GLU VAL LYS ALA ALA LEU LYS GLU ASP ALA LEU
   COORDS: GLY GLY LEU GLU GLU VAL LYS ALA ALA LEU LYS GLU ASP ALA LEU
           46                                                          60

           61                                                          75
   SEQRES: GLN PHE ALA TYR TYR ARG THR ILE SER GLY ASP GLU GLU SER LYS
   COORDS: GLN PHE ALA TYR TYR ARG THR ILE SER GLY ASP GLU GLU SER LYS
           61                                                          75

           76                                                          90
   SEQRES: ARG VAL LYS PHE VAL PHE ILE SER TRP ALA GLY GLU GLY ILE LYS
   COORDS: ARG VAL LYS PHE VAL PHE ILE SER TRP ALA GLY GLU GLY ILE LYS
           76                                                          90

           91                                                         105
   SEQRES: LYS PRO LYS LEU ARG ALA VAL MSE SER ILE LEU LYS GLY ASP VAL
   COORDS: LYS PRO LYS LEU ARG ALA VAL MSE SER ILE LEU LYS GLY ASP VAL
           91                                                         105

           106                                                        120
   SEQRES: LYS ASN VAL ILE ASN ASN PHE HIS ILE GLU LEU HIS ALA THR SER
   COORDS: LYS ASN VAL ILE ASN ASN PHE HIS ILE GLU LEU HIS ALA THR SER
           106                                                        120

           121                                                        135
   SEQRES: LEU ASP ASP LEU VAL GLU ASP GLU ILE ALA ALA LYS ILE LYS LEU
   COORDS: LEU ASP ASP LEU VAL GLU ASP GLU ILE ALA ALA LYS ILE LYS LEU
           121                                                        135

           136                     142
   SEQRES: GLU HIS HIS ALA HIS HIS HIS
   COORDS: GLU HIS HIS ALA   ?   ?   ?
           136         139
```
